# Position of catheter‐through‐needle erector spinae plane catheters for rib fracture analgesia assessed by computed tomography

**DOI:** 10.1002/anr3.70079

**Published:** 2026-06-21

**Authors:** B. Goulson, F. Malik, N. Suarez, M. Luney

**Affiliations:** ^1^ Nuffield Department of Anaesthesia Oxford University Hospitals NHS Foundation Trust Oxford UK; ^2^ Department of Radiology Oxford University Hospitals NHS Foundation Trust Oxford UK; ^3^ Nuffield Department of Clinical Neurosciences University of Oxford Oxford UK; ^4^ Kadoorie Institute, Nuffield Department of Orthopaedics, Rheumatology and Musculoskeletal Sciences University of Oxford Oxford UK

**Keywords:** catheter displacement, catheter‐through‐needle, computed tomography, erector spinae plane block, rib fractures

## Abstract

Erector spinae plane catheters are increasingly used for rib fracture analgesia, but the influence of catheter insertion technique on catheter position and analgesic effectiveness remains uncertain. In this case series, we describe 11 erector spinae plane catheters inserted using a catheter‐through‐needle technique for analgesia in patients with rib fractures who underwent chest computed tomography after catheter insertion. Catheter tip position outside the intended fascial plane was identified in six of 11 (55%) catheters. Previous studies of catheter‐over‐needle systems have reported displacement rates of up to 89%, but cross‐study comparisons are limited by small sample sizes and differences in population, catheter techniques and imaging protocols. Interpretation of clinical impact is limited. Pain score recording and analgesic prescribing were not standardised, and patients frequently had multiple injuries requiring multimodal analgesia, making it difficult to isolate the contribution of erector spinae planes catheter position to pain relief. Furthermore, computed tomography demonstrates catheter location but may not reflect local anaesthetic spread or functional block efficacy. These findings highlight clinically relevant uncertainty regarding the relationship between catheter insertion technique, catheter position and patient‐centred analgesic effectiveness. Further prospective studies are needed to determine whether insertion techniques affect catheter position, injectate spread and patient‐centred analgesic outcomes.

## Introduction

Rib fractures are associated with significant morbidity and mortality, largely due to impaired ventilation, atelectasis and pneumonia, which may be mitigated by effective analgesia [1]. Continuous erector spinae plane (ESP) catheters are increasingly used to provide analgesia for rib fractures and reduce opioid consumption [2]. Their effectiveness may depend on accurate placement within the intended fascial plane and maintenance of this position over time [3]. However, data on post‐insertion ESP catheter position are limited, as cross‐sectional imaging after catheter placement is uncommon.

A recent study reported high rates of catheter displacement using catheter‐over‐needle (CON) systems, with displacement observed in up to 89% of cases [4]. The standard insertion technique at our institution is a catheter‐through‐needle (CTN) technique. This technique may offer theoretical advantages, including greater catheter length within the plane and more favourable insertion angles, which could reduce displacement. However, comparable data on computed tomography (CT)‐assessed catheter position after CTN insertion are lacking.

We therefore aimed to assess the position of CTN ESP catheters on subsequent CT imaging in patients receiving rib fracture analgesia and to consider how these findings might inform future comparative studies.

## Methods

This single‐centre case series was conducted at the John Radcliffe Hospital, Oxford, UK. Patients were eligible, if they were aged ≥ 18 years with rib fractures and one or more ESP catheters inserted. Patients were excluded, if they did not undergo chest CT scan within 120 hours of catheter insertion or if the catheter had been inserted at another hospital. For governance purposes, the UK Health Research Authority decision tool classified this project as a service evaluation rather than research; therefore, Research Ethics Committee review and individual patient consent were not required. We obtained Caldicott Guardian approval as required by our institutional policy. Participants were identified from the acute pain service electronic referrals and data extracted from the electronic health record. To align with existing literature, we recorded CT slice thickness, catheter position and the transverse and anteroposterior distances from the catheter tip to the ESP [4]. The primary outcome was the proportion of catheter tips located outside the ESP on CT imaging. The secondary outcome was catheter distance from the ESP.

Erector spinae plane catheters were inserted using a CTN technique with a 50 or 100 mm 18G Tuohy needle (SonoLong Echo Nanoline^®^, Pajunk, Gersingen, Germany), using an in‐plane, high‐frequency linear‐array ultrasound probe technique. After hydro‐dissection with normal saline or local anaesthetic, catheters were advanced 3–5 cm within the plane. Catheters were secured using skin glue, clear adhesive dressings and self‐adhesive fabric tape.

## Results

Five hundred and eleven patients with rib fractures were referred to the acute pain service between 01/10/2023 and 28/11/2025, of whom 122/511 (23.9%) received an ESP catheter. Ten patients had available CT imaging. The median (IQR) age was 70 (58–77) years, and 6/10 (60%) were female. One patient had bilateral fractures with one catheter inserted on each side. The mean (SD) number of fractured ribs per affected side was 6.54 (1.87). The mean (SD) time from catheter insertion to scan was 44.7 (28.3) hours and catheters remained in situ for a mean (SD) of 5 (0.98) days. All catheters were removed within eight days.

On CT imaging, catheter tip position outside the ESP was identified in 6/11 (55%) of catheters. Five catheter tips were intramuscular and one was subcutaneous (Table 1). Among the catheter tips outside the ESP, the median (IQR [range]) transverse distance from the catheter tip to the intended plane was 0.5 (0–20 [0–27]) mm and the median anteroposterior distance was 9.5 (7–14 [0–18]) mm.

**Table 1:** Computed tomography‐assessed position of individual erector spinae plane catheters.

Catheter	Depth at skin, cm	Fracture side	Fractured ribs, n	ESP catheter side	Time to CT imaging, days	Catheter tip within ESP	Catheter tip location	Transverse distance to ESP, mm	AP distance to ESP, mm
1	11	Right	10	Right	1	Yes	ESP	0	0
2A	6	Bilateral	7	Left	1	No	IM	1	11
2B	8	Bilateral	7	Right	1	No	IM	0	18
3	11.5	Left	7	Left	3	No	IM	0	15
4	12	Left	7	Left	1	No	IM	0	7
5	11	Right	7	Right	1	Yes	ESP	0	0
6	13	Left	6	Left	0	Yes	ESP	0	0
7	11	Left	8	Left	2	Yes	ESP	0	0
8	11	Left	7	Left	3	No	SC	26	8
9	15	Left	8	Left	5	Yes	ESP	0	0
10	12	Left	5	Left	4	No	IM	27	0

CT, computed tomography; ESP, erector spinae plane; AP, anteroposterior; IM, intramuscular; SC, subcutaneous.

Pain scores at rest were available for all patients. On a 0–3 pain scale (0, no pain; 1, mild pain; 2, moderate pain; 3, severe pain), median pain scores in the 24 hours preceding catheter insertion ranged from 1 to 2. Following catheter placement, pain scores remained between 0 and 2 from day 0 to day 4 after ESP catheter insertion. There was no clear difference in pain trajectories between patients with catheter tips positioned within and outside the ESP.

## Discussion

We found that the catheter tip was outside the ESP in 55% of catheters inserted using a CTN technique on subsequent CT imaging. Previous studies of CON systems have reported catheter displacement in up to 89% of cases [4]. The lower proportion observed in this CTN series is hypothesis‐generating and cross‐study comparisons are limited by small sample sizes, differences in patient populations, catheter techniques and imaging protocols.

The observed difference may relate to technical factors, such as the longer needle and shallower angle of approach used in CTN technique or the greater length of catheter inserted. Median depth of catheter at skin was 11 cm, compared to 6.2 cm reported in a CON cohort [4]. However, these explanations remain speculative and require prospective evaluation.

Interpretation of pain scores was limited, with no consistent relationship observed between catheter position and analgesic outcomes. Several explanations may account for this. First, all patients in this cohort had injuries to other anatomical sites, and all patients received multimodal analgesia including systemic opioids, making it difficult to isolate the effect of ESP catheter performance. Second, local anaesthetic may still spread within the paraspinal fascial compartments even when the catheter tip lies outside the plane. Computed tomography provides only a static assessment of catheter position and does not capture dynamic local anaesthetic spread. As such, catheter location alone may be an incomplete surrogate for block efficacy. Erector spinae plane blocks are known to have variable dermatomal spread, for which routine clinical assessment (sensation to cold testing) may be impractical, further limiting interpretation of block efficacy [5].

This study has several limitations. First, it is a case series with a small sample size and single‐centre design. Second, selection bias is likely, as only patients who underwent CT imaging after catheter insertion were included, which may not represent the wider population receiving ESP catheters. In addition, CT imaging was not performed at a uniform time point, and it is therefore not possible to determine whether catheters were initially placed outside the intended fascial plane or migrated between insertion and imaging. Finally, CT provides anatomical rather than functional information and cannot assess local anaesthetic spread or block efficacy directly.

In conclusion, catheter tip position outside the intended fascial plane was identified in 55% of CTN ESP catheters on subsequent CT imaging. Although this proportion is lower than previously reported for CON systems, cross‐study comparisons are limited, and the findings should be interpreted as hypothesis‐generating. Future prospective studies comparing CTN and CON techniques should assess whether insertion technique influences catheter position, injectate spread and patient‐centred outcomes including pain scores, opioid use, functional outcomes and pulmonary complications.
